# DENV-1 Genotype V in Brazil: Spatiotemporal dispersion pattern reveals continuous co-circulation of distinct lineages until 2016

**DOI:** 10.1038/s41598-018-35622-x

**Published:** 2018-11-21

**Authors:** Fernanda de Bruycker-Nogueira, Thiara Manuele Alves Souza, Thaís Chouin-Carneiro, Nieli Rodrigues da Costa Faria, Jaqueline Bastos Santos, Maria Celeste Torres, Izabel Letícia Cavalcante Ramalho, Shirlei Ferreira de Aguiar, Rita Maria Ribeiro Nogueira, Ana Maria Bispo de Filippis, Flavia Barreto dos Santos

**Affiliations:** 1 Viral Immunology Laboratory, Oswaldo Cruz Institute - FIOCRUZ, Rio de Janeiro, Brazil; 2 Flavivirus Laboratory, Oswaldo Cruz Institute - FIOCRUZ, Rio de Janeiro, Brazil; 3 Central Laboratory of Public Health of Ceará - LACEN-CE, Fortaleza, Brazil; 4 Central Laboratory of Public Health of Rio de Janeiro - LACEN-RJ, Rio de Janeiro, Brazil

## Abstract

In Brazil, DENV-1 introduced in the 80’s, remained the prevalent serotype from 2012 to 2016. After its re-emergence in the country in 2009, the co-circulation of different viral lineages was identified, however, its transmission dynamics afterwards, was not fully characterized. In this study, we performed the continuous molecular surveillance after the reemergence period (2012 to 2016), covering the 30 years of circulation of DENV-1 in Brazil. Phylogenetic analysis allowed confirmation of the continued presence of genotype V, as well as three distinct co-circulating lineages. The molecular characterization of the E gene presented two new amino acid substitutions previously unidentified in the country. Phylogeographic analysis has shown that a large flow of migrations has occurred between Brazil and Argentina in the last 10 years.

## Introduction

The dengue virus (DENV) belongs to the family *Flaviviridae*, genus *Flavivirus*, and presents four serotypes antigenically distinct, DENV-1 to DENV-4^1^. They are classified as arboviruses, since its maintenance in nature occurs through a transmission cycle of vertebrate hosts and hematophagous arthropods^2^, most prevalently in tropical and subtropical areas worldwide^3^.

Dengue is one of the main public health problems in the world, with relevant social and economical impact due to the increased geographic extension, number of cases and disease severity^4^ and, in Brazil, DENV activity has grown significantly since the introduction of DENV-1 in Rio de Janeiro (RJ) in 80’s. This serotype re-emerged in 2009 after approximately eight years without being related to epidemics, causing in 2010, more than one million probable cases, with the highest hospitalization rate reported in the country, especially in those over 60 years old. In the last 32 years, more than twelve million dengue cases have been reported in Brazil^5,6^.

After reemergence period (2009–2010), the DENV-1 and DENV-4 were responsible for a high number of cases in the following years, reviewed in dos Santos *et al*.^7^, being the DENV-1 prevalent in years of 2014 to 2016, representing proximally 90% of isolated cases in country^8,9^. This serotype continue to be identified until today^10^.

Phylogenetic analysis of DENV-1 since its introduction in the country, characterized those viruses as belonging to genotype V, with its origin in the Caribbean and Venezuela and showed that several independent introductions occurred over time with high genotype variability^11–17^.

Molecular characterization and evolutionary studies can be important tools to monitor the introduction and understanding of the viruses spread in a region, as well as to predict possible epidemiological consequences of such events. In this scenario, the DENV serotypes surveillance is of great relevance, as it may provide information on the spread of potentially virulent strains, as well as, to assess their impact on the population during an outbreak. Here, we performed the molecular characterization and investigated the spatiotemporal patterns of DENV-1 lineages emergence and dissemination in Brazil during 30 years, including strains from 2012 to 2016, representative of its post-reemergence and intense circulation in the country.

## Results

### Phylogenetic analysis of DENV-1 strains based on Envelope gene

**Figure 1:**
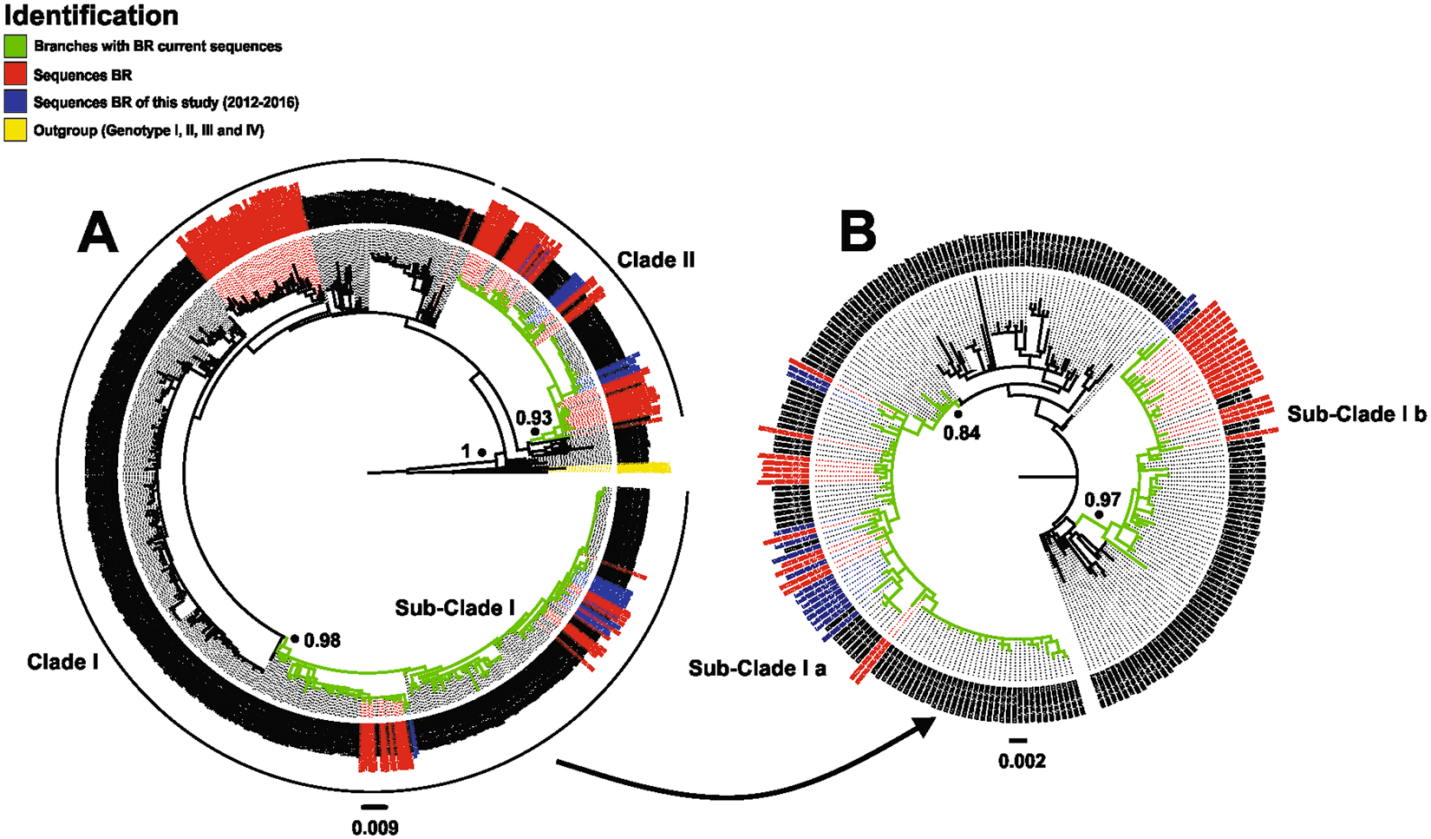
ML tree of E gene sequences of DENV-1 Genotype V. The aLRT support values of major branching are shown. (**A**) ML tree of 751 E gene sequences. (**B**) ML tree of 240 E gene sequences of Sub-Clade I.

For phylogenetic analysis of current DENV-1 lineages isolated from patients from 2012 to 2016 in Brazil, the envelope (E) gene sequencing of viral strains (*n* = 30), of four distinct geographic region: Southeast (*n* = 20), Central-Western (n = 4), North (*n* = 1) and Northeast (n = 5) (Table 1), was performed. The Maximum Likelihood (ML) phylogenetic analysis of 717 E gene sequences of DENV-1 genotype V available in Genbank, additionally to thirty sequences generated in this study, and sequences representing each DENV-1 genotype (I to IV), totalizing 751 sequences, showed that all strains currently belong to genotype V, but grouped in two distinct clades, previously identified by the our group, as Clade I and Clade II into the cosmopolitan clade of genotype V (Fig. 1A).

**Table 1:** DENV-1 strains (n = 30) used in this study for envelope (E) gene (1,485 nucleotides) sequencing, 2012 to 2016, Brazil.

ID samples	Year	Country region: state	Origin of strain	Genbank Accession number
2071/2012/BR/RJ/2012	2012	Southeast: RJ	Isolated (C6/36)	MH401971
2612/2012/BR/RJ/2012	2012	Southeast: RJ	Isolated (C6/36)	MH401972
3239/2012/BR/RJ/2012	2012	Southeast: RJ	Isolated (C6/36)	MH401973
3246/2012/BR/RJ/2012	2012	Southeast: RJ	Isolated (C6/36)	MH401974
3599/2012/BR/RJ/2012	2012	Southeast: RJ	Isolated (C6/36)	MH311981
Lac1/BR/RJ/2012	2012	Southeast: RJ	Serum	MH401990
Lac5/BR/RJ/2012	2012	Southeast: RJ	Serum	MH401991
Lac16/BR/RJ/2012	2012	Southeast: RJ	Serum	MH401992
92/2013/BR/RJ/2013	2013	Southeast: RJ	Isolated (C6/36)	MH401977
7276/2013/BR/MS/2013	2013	Midwest: MS	Serum	MH401975
7436/2013/BR/MS/2013	2013	Midwest: MS	Serum	MH401976
05/2014/BR/RJ/2014	2014	Southeast: RJ	Isolated (C6/36)	MH401978
478/2014/BR/RJ/2014	2014	Southeast: RJ	Isolated (C6/36)	MH401979
494/2014/BR/SE/2014	2014	Northeast: SE	Isolated (C6/36)	MH401980
283/2017/BR/CE/2014	2014	Northeast: CE	Serum	MH401987
288/2017/BR/CE/2014	2014	Northeast: CE	Serum	MH401988
297/2017/BR/CE/2014	2014	Northeast: CE	Isolated (C6/36)	MH401989
30/2015/BR/RJ/2015	2015	Southeast: RJ	Isolated (C6/36) (passage 1)	MH401981
134/2015/BR/RJ/2015	2015	Southeast: RJ	Isolated (C6/36) (passage 1)	MH401982
148/2015/BR/SE/2015	2015	Northeast: SE	Isolated (C6/36)	MH401986
738/2015/BR/RJ/2015	2015	Southeast: RJ	Isolated (C6/36)	MH401983
2072/2015/BR/RJ/2015	2015	Southeast: RJ	Isolated (C6/36)	MH401984
3171/2015/BR/RJ/2015	2015	Southeast: RJ	Isolated (C6/36)	MH401985
Lac7/BR/RJ/2015	2015	Southeast: RJ	Serum	MH401993
67/2015/BR/AP/2015	2015	North: AP	Serum	MH401997
Lac4/BR/RJ/2016	2016	Southeast: RJ	Serum	MH401994
Lac8/BR/RJ/2016	2016	Southeast: RJ	Isolated (C6/36)	MH401995
Lac9/BR/RJ/2016	2016	Southeast: RJ	Serum	MH401996
KCMM25/BR/MS/2016	2016	Midwest: MS	Serum	MH401998
VAOR28/BR/MS/2016	2016	Midwest: MS	Serum	MH401999

ID: Identification; AP: Amapá; BR: Brazil; CE: Ceará; MS: Mato Grosso do Sul; RJ: Rio de Janeiro; SE: Sergipe.

In order to analyze the branches that actually had recent Brazilian sequences, we separated from Clade I, the subset corresponding to Sub-Clade I, containing 240 sequences and submitted to a new ML analysis to identify the specific branches with Brazilian representatives (Fig. 1B). From this analysis, three subsets were separated for phylogeographic reconstruction: Sub-Clade Ia (n = 106), Sub-Clade Ib (n = 56) and Clade II (n = 116) (Table S2).

### Dispersion of Sub-Clade Ia and Ib of DENV-1 genotype V in Brazil

The Sub-Clade I was constituted with 240 sequences representative of 11 countries with 1 to 66 sequences each, include current Brazilian strains (n = 48) from four locations. The Brazilian sequences were distributed according the geographic regions as follows: Midwest (*n* = 4), Southeast (*n* = 18), Northeast (*n* = 7) and North (*n* = 19) (Table S2, Sub-Clade I). Moreover, the Sub-Clades Ia and Ib identified within this Sub-Clade were submitted separately to a Bayesian phylogeographic analysis.

**Figure 2:**
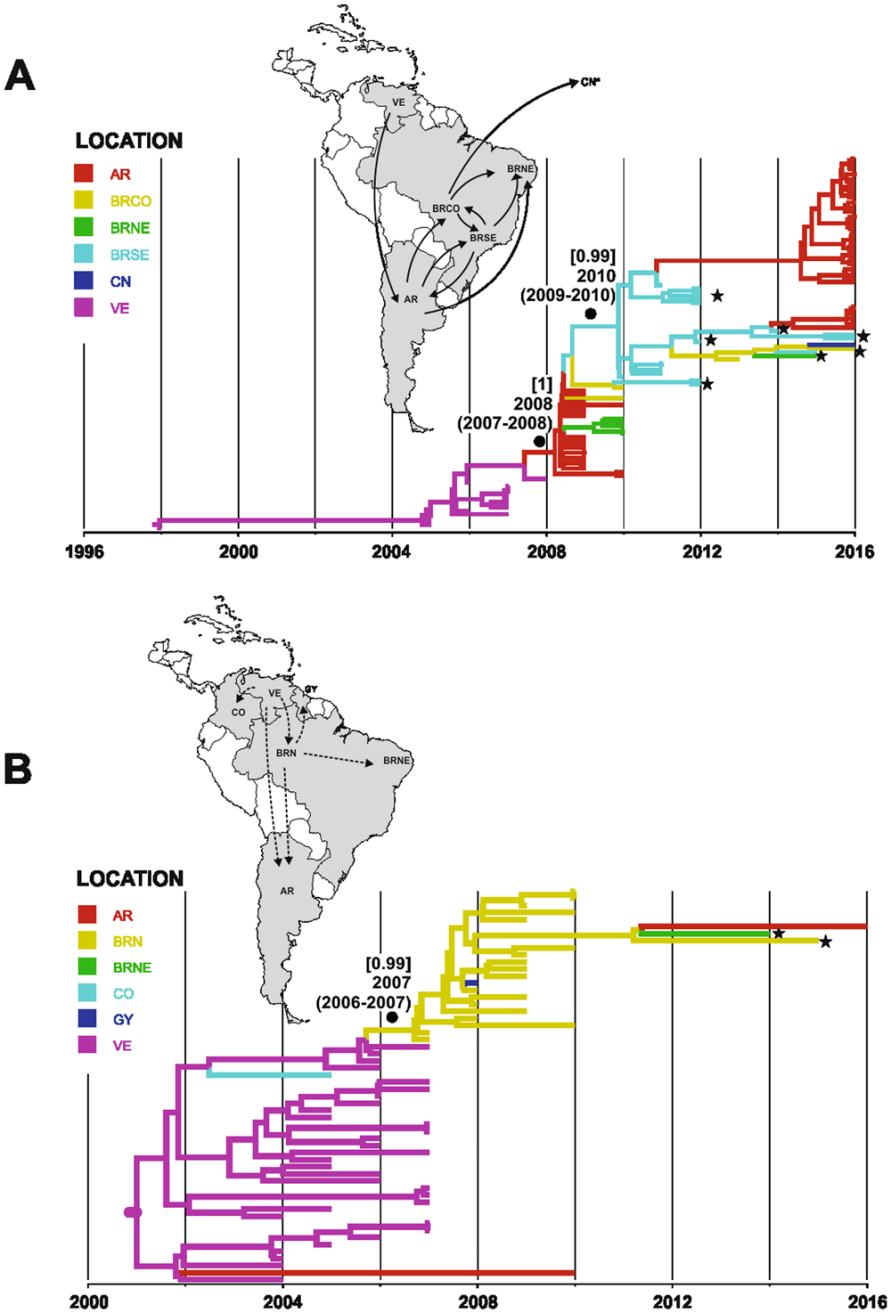
Time-scaled Bayesian Maximum Clade Credibility tree for the Sub-Clade Ia and Ib of E gene DENV-1 Genotype V. (**A**) Sub-Clade Ia (n = 106 sequences). (**B**) Sub-Clade Ib (n = 56 sequences). Branches are colored according to the most probable location (legend shown on the left side) of their parental node inferred by discrete phylogeographical analysis. Posterior probability - *PP* values in [] and T_MRCA_ (95% HPD) are represented in node the of branches. All horizontal branch lengths are drawn to a scale of years. The tree is automatically rooted under the assumption of a relaxed molecular clock. AR: Argentina; CO: Colombia; GY; French Guyana; VE: Venezuela; CN: China; BRN: North of Brazil; BRNE: Northeast of Brazil; BRSE: Southeast of Brazil; BRCO: Central-Western of Brazil. Viral dispersal pattern are showed in map. Lines between locations represent branches in the Bayesian MCC tree along which location transitions occurs. Star symbol: samples this study.

The Sub-Clade Ia analysis showed that DENV-1 strains reached Argentina around 2008 from Venezuela (posterior state probability [PSP] = 0.99). From Argentina this clade spread to Brazil, to the Northeast, Midwest and Southeast regions. This viral variant remained circulating until after reaching the Southeast region approximately in 2010, coming from Argentina [PSP = 0.94]. Since then, introductions in the Midwest, Northeast and Argentina have occurred. An interesting spread was observed from the Midwest region to China [PSP = 0.7] around the year 2015 (Fig. 2A).

Sub-Clade Ib presented an introduction route in North Brazil region directly from Venezuela around 2007 [PSP = 0.99] and it seems to have remained circulating only in the North of the country, with a point-scattered event for the French Guiana in 2008 [PSP = 0.99] and in 2012, for Argentina and the Northeast of the country [PSP = 0.57] (Fig. 2B).

### Dispersion of Clade II of DENV-1 genotype V in Brazil

The Clade II comprised the Subset-Clade II, containing 116 sequences from four countries, including Brazil, with 67 sequences divided into four locations, and represented by 1 to 46 sequences each. The Brazilian sequences were distributed according to geographic regions as follows: Midwest (n = 17), Southeast (n = 21), Northeast (n = 10) and North (n = 19) (Table S2, Subset-Clade II).

**Figure 3:**
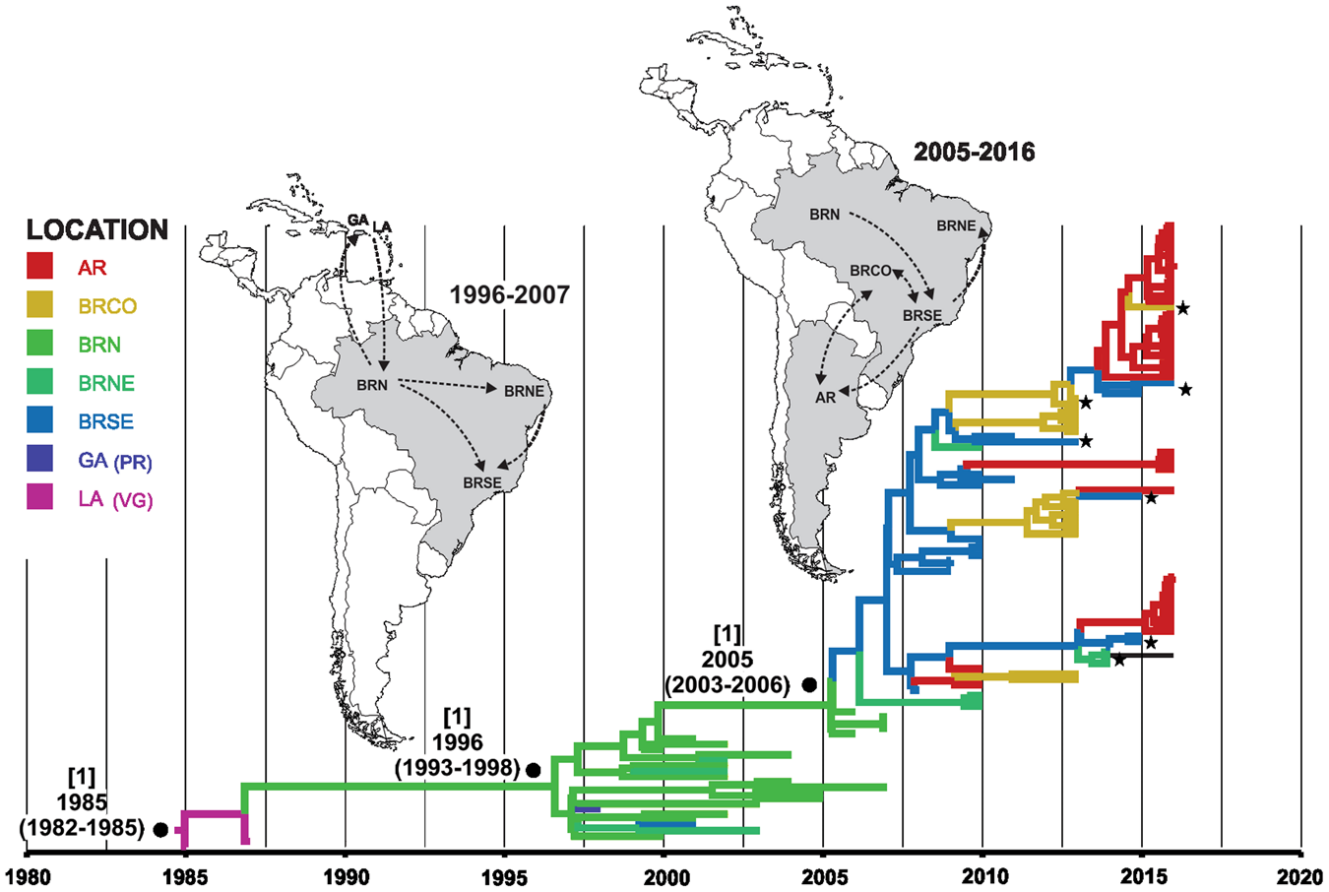
Time-scaled Bayesian Maximum Clade Credibility tree for the 116 sequences of Clade II of E gene DENV-1 Genotype V. Branches are colored according to the most probable location (legend shown on the left side) of their parental node inferred by discrete phylogeographical analysis. Posterior probability - *PP* values in [] and T_MRCA_ (95% HPD) are represented in node the of branches. All horizontal branch lengths are drawn to a scale of years. The tree is automatically rooted under the assumption of a relaxed molecular clock. AR: Argentina; GA (PR): Greater Antilles - Puerto Rico; LA (VG): Lesser Antilles - Virgins Island; BRN: North of Brazil; BRNE: Northeastern of Brazil; BRSE: Southeast of Brazil; BRCO: Central-Western of Brazil. Viral dispersal pattern are showed in map. Lines between locations represent branches in the Bayesian MCC tree along which location transitions occur. Star symbol: samples this study.

The subset was also subjected to a Bayesian phylogeographic analysis and the results showed that this subset appears to have arisen in the Caribbean Lesser Antilles (LA), represented here, by sequences from the Virgin Islands (VG), in the mid-1980s [PSP = 0.97] and remained without identified circulation until 2000. This clade arrived in Brazil in the mid-1990s [PSP = 0.95] by the North region and spread for that region until the mid-2000s, with occasional migration events to the Greater Antilles (GA) (represented here by the Puerto Rico [PR] strain) and to Northeast and Southeast regions of country. However, around 2005 and 2006-7, this clade arrived in the Southeast [PSP = 0.99] and Northeast regions [PSP = 0.78], respectively. From the Southeast region the clade spread to two directions: (1) For Argentina [PSP = 0.99] and then for Midwest [PSP = 0.63], for Argentina in two other moments [PSP = 0.78 and 0.98] and for Northeast [PSP = 0.98] and, (2) For Northeast [PSP = 0.97], Argentina [PSP = 1] and Midwest [PSP≥ 0.94]. From the Midwest, this clade spread for the Southeast region of Brazil and Argentina, then back to the Midwest region again [PSP = 1]. A large flow of migrations between the Southeast, Midwest and Argentina seems to be occurring since 2010. Of the 30 sequences generated in this study, fourteen representing the Southeast, Midwest and Northeast regions of Brazil form 2013 to 2016, belong to this clade and were involved in this migration dynamics (Fig. 3).

### Molecular characterization of DENV-1 strains based on E gene

The sequences alignment demonstrated different patterns of nucleotides responsible for conserving some amino acid in domains I, II, III and stem region of the E gene among the strains of the different clusters. In order to identified those differences, we used a strain from the period of DENV-1 introduction in Brazil, for comparison purposes (Table 2).

**Table 2:** Amino acid conserved of the distinct Brazilian DENV- 1 genotype V lineages based on analysis of the envelope (E) gene.

ID/country/state/year	E gene (position)/substitution of amino acid							Clade cosmopolitan (Genotype-V)* Clade
	Domain II	Domain I	Domain III			Stem		
	E_230_	E_297_	E_338_	E_361_	E_394_	E_428_	E_436_	
HQ026760/BR/RJ/1986	**T**	**T**	**S**	**K**	**K**	**V**	**V**	Sub-Clade I BR*
2071/2012/BR/RJ/2012	.	**M**	.	.	**R**	.	.	Sub-Clade I a
2612/2012/BR/RJ/2012	.	**M**	.	.	**R**	.	.	Sub-Clade I a
3239/2012/BR/RJ/2012	.	**M**	.	.	**R**	.	.	Sub-Clade I a
3246/2012/BR/RJ/2012	.	**M**	.	.	**R**	.	.	Sub-Clade I a
3599/2012/BR/RJ/2012	.	**M**	.	.	**R**	.	.	Sub-Clade I a
05/2014/BR/RJ/2014	.	**M**	.	.	**R**	.	.	Sub-Clade I a
148/2015/BR/SE/2015	.	**M**	.	.	**R**	.	.	Sub-Clade I a
Lac1/BR/RJ/2012	.	**M**	.	.	**R**	.	.	Sub-Clade I a
Lac5/BR/RJ/2012	.	**M**	.	.	**R**	.	.	Sub-Clade I a
Lac16/BR/RJ/2012	.	**M**	.	.	**R**	.	.	Sub-Clade I a
Lac7/BR/RJ/2015	.	**M**	.	.	**R**	.	.	Sub-Clade I a
Lac4/BR/RJ/2016	.	**M**	.	.	**R**	.	.	Sub-Clade I a
Lac8/BR/RJ/2016	.	**M**	.	.	**R**	.	.	Sub-Clade I a
VAOR28/BR/MS/2016	.	**M**	.	.	**R**	.	.	Sub-Clade I a
297/2017/BR/CE/2014	**A** ^**#**^	**M**	.	.	.	.	.	Sub-Clade I b
67/2015/BR/AP/2015	**A** ^**#**^	**M**	.	.	.	.	.	Sub-Clade I b
92/2013/BR/RJ/2013	.	**M**	**L**	.	.	**L**	**I**	Clade II
7276/2013/BR/MS/2013	.	**M**	**L**	.	.	**L**	**I**	Clade II
7436/2013/BR/MS/2013	.	**M**	**L**	.	.	**L**	**I**	Clade II
134/2015/BR/RJ/2015	.	**M**	**L**	.	.	**L**	**I**	Clade II
2072/2015/BR/RJ/2015	.	**M**	**L**	.	.	**L**	**I**	Clade II
3171/2015/BR/RJ/2015	.	**M**	**L**	.	.	**L**	**I**	Clade II
KCMM25/BR/MS/2016	.	**M**	**L**	.	.	**L**	**I**	Clade II
Lac9/BR/RJ/2016	.	**M**	**L**	.	.	**L**	**I**	Clade II
283/2017/BR/CE/2014	.	**M**	**L**	**R**	.	**L**	**I**	Clade II
288/2017/BR/CE/2014	.	**M**	**L**	**R**	.	**L**	**I**	Clade II
478/2014/BR/RJ/2014	.	**M**	**L**	**R**	.	**L**	**I**	Clade II
494/2014/BR/SE/2014	.	**M**	**L**	**R**	.	**L**	**I**	Clade II
30/2015/BR/RJ/2015	.	**M**	**L**	**R**	.	**L**	**I**	Clade II
738/2015/BR/RJ/2015	.	**M**	**L**	**R**	.	**L**	**I**	Clade II

T: threonine; S: serine; K: lysine; V: valine; A: alanine; M: methionine; L: leucine; R: arginine; I: isoleucine. ^*^Classified according de Bruycker-Nogueira et al., 2016. ^#^In these samples and one reference of the Argentina (KX768377). In others sequences of this clade the amino acid found is Threonine (T).

In domain I, at position E_297_, the amino acid methionine (M) was observed in all 30 sequences studied, different from the 80’s sequence which has a threonine (T) at that same position. In domain II, we identified the Alanine (A) at position E_230_ in only two of our sequences studied, belonging to the Sub-Clade Ib. All other sequences have a T at this position. Analyzing all 240 sequences of the Sub-Clade I, only those two sequences and one from Argentina from 2016 (KX768377), presented this amino acid.

Further conserved amino acids were observed in the domain III (E_338_, E_361_ and E_394_) and stem region (E_428_ and E_436_). The sequences from Sub-Clade Ia presented in E_394_ an Arginine (R) in the place of Lysine (K) like the other sequences of Sub-Clade Ib and Clade II. However, Clade II sequences showed the greatest differences between shared amino acids, with a Leucine (L) at E_338_ and E_428_ and an Isoleucine (I) at E_436_, amino acids conserved in all clades. Additionally, six sequences from this study from 2014 and 2015, representative from the Southeast and Northeast regions, share an R in E_361_ with eleven sequences from Argentina from 2016, differentiating those from the other sequences from Clade II and Sub-Clade I dataset, which have a K at the same position.

## Discussion and Conclusion

The Sub-Clade Ia had Argentina as the gateway to Brazil, spreading to the Northeast, Midwest and Southeast regions of the country. The observation of Argentina as an ancestor for the Brazilian strains, corroborate our previous study^15^. With the introduction in the Southeast region, we observed most of the dispersions of the viral strain within Brazil, as well as returning to Argentina, which circulated at least until 2016.

The viral spread from the Midwest region to China, probably refers to an imported case. At Genbank, that Chinese strain does refer to an imported case study, but it has not been published. However, the results obtained here suggest this probable importation from Brazil, more specifically, from the Midwest region.

Sub-Clade Ib presented an introduction route in Northeast Brazil starting directly from Venezuela, as already demonstrated previously^15^. This clade seems to have remained circulating only in the North of the country, with a punctual dispersal event for the French Guianas. However, we observed a more recent movement of this clade to the Northeast region of Brazil and to Argentina. Tittarelli and collaborators studying isolates from Argentina during the epidemic that occurred in early 2016 highlights that a sample of their study was related to samples from the North region of Brazil^18^.

The Clade II migrations occurred until the mid-2000s after reaching the Southeast region of the country. From the Southeast region, this clade dispersed directly to Argentina, to the Northeast and also remained in the Southeast region. It reached the Midwest directly through the Southeast and through Argentina, migrating again from this to the Midwest. A large flow of migrations between the Southeast, the Midwest and Argentina seems to be occurring during the decade of 2010.

The molecular characterization of the 30 strains allowed the identification of different nucleotides patterns responsible for the conservation of some amino acids in the domains I, II, III and stem-region of the E gene among the different strains and the changes in domain II and one in domain III, were not previous identified. In domain I, at position E_297_, the amino acid M was observed in all 30 sequences studied, different from the sequence of the 80s. In domain II, we identified an A at position E_230_ in only two of our studied sequences, belonging to Sub-Clade Ib. All other sequences have a T at this position. Analyzing all 240 sequences of Clade I, only those two sequences and one from Argentina 2016 had this amino acid. During a study conducted in Argentina from December 2015 to April 2016, this amino acid was not reported^18^. In fact, in all the dataset analyzed here, the only sequences that present this shared amino acid are two of our study detected in 2014 and 2015 and the one from Argentina, detected afterwards in that country. Preserved amino acids were observed in domain III and in the stem region. The sequences of Sub-Clade Ia presented in E_394_ an R instead of K, like the other sequences of Sub-Clade I and Clade II. However, Clade II sequences showed the largest differences among shared amino acids.

Recently, Dutra and collaborators investigated circulating DENV-1 strains in an epidemic in the state of Minas Gerais, and reported the co-circulation of two distinct lineages in 2013 and five independent introductions of genotype V in the country since 1982^19^. The nomenclature of the different DENV-1 lineages circulating in Brazil is yet to be defined. In our study, we designated the clades with roman numerals I and II, and letters for the lineages within a clade. However, Dutra *et al*.^19^ suggest the standardization of BR1 to BR5 to the lineages, according to the chronological order of introduction in country.

In conclusion, our data show that DENV-1 remains circulating in the country through three distinct lineages, introduced by independent pathways in the last two decades. One of the lineages seems to be restricted to the North region of the country, while the other two are more dispersed by Northeast, Southeast and Midwest. Continuous surveillance is necessary, as new viral variants may arise by local evolution or by introductions from other countries. New viral strains may present nucleotide and/or amino acid changes in viral genome important for infection and replication, impacting the dynamics of disease maintenance and transmission.

## Materials and Methods

### Ethical statement

The strains analyzed in this study belong to a previously gathered collection from the Flavivirus Laboratory, IOC/FIOCRUZ, Rio de Janeiro, Brazil, obtained from human serum from an ongoing Project approved by resolution number CSN196/96 from the Oswaldo Cruz Foundation Ethical Committee in Research (CEP 274/05) and collections during a cross-sectional and observational study performed by the Viral Imunnology Laboratory, IOC/FIOCRUZ, Rio de Janeiro, Brazil approved by the Oswaldo Cruz Foundation Ethic Committee (CAAE 57221416.0.1001.5248). The informed consent was obtained from all subjects. Samples were chosen anonymously, based on the laboratorial results and clinical manifestations available on the Laboratory database. All methods were performed in accordance with relevant guidelines and regulations.

### Dengue viral strains

The DENV-1 strains (*n* = 30) analyzed were detected in serum samples from patients positive for dengue by virus isolation and/or RT-PCR, received at the Flavivirus Laboratory (LABFLA), IOC/FIOCRUZ, Regional Reference Center for Dengue and Yellow Fever Diagnosis and Viral Immunology Laboratory (LIV), IOC/FIOCRUZ. Viral strains were selected according to the year of isolation and state of origin. Fifteen strains were derived of the original isolate on cell culture, two after one passage of the original isolate on cell culture and thirteen were analyzed directly from the serum sample (Table 1). Virus isolation was performed by inoculation into C6/36 *Aedes albopictus* cell line^20^ and isolates were identified by indirect fluorescent antibody test (IFAT) using serotype-specific monoclonal antibodies^21^.

### Reverse transcription followed by the polymerase chain reaction (RT-PCR)

Viral RNA was extracted from 140 μL of supernatant from cultures isolated or serum using the QIAmp Viral Mini Kit (Qiagen, Inc., Germany) according to the protocol described by the manufacturer and stored at −70 °C. The methodology described by Lanciotti *et al*.^22^ that detects all four serotypes simultaneously in a semi-nested procedure, generating amplification products with specific size in base pairs (bp) of each DENV serotype, was used to confirm the DENV-1 strains positivity.

### Dengue virus genome amplification and sequencing

For E gene sequencing of DENV-1, three primers pairs (sets 2–4) were used to amplify overlapping fragments of approximately 900 bp according as previously described by our group^16^. Sequencing was performed on an ABI 3730 DNA Analyzer, Applied Biosystems®, California, USA^23^ and the sequences generated were deposited on GenBank (Table 1). The sequences’ analysis was performed using the Bioedit (http://www.mbio.ncsu.edu/bioedit/bioedit.html), the sequences’ identity by BLAST (http://blast.ncbi.nlm.nih.gov/Blast.cgi) and alignments by CLUSTAL OMEGA (https://www.ebi.ac.uk/Tools/msa/clustalo/).

### Sequences dataset

Complete E gene (1485 bp) sequences of DENV-1 genotype V (Cosmopolitan clade) with known sampling date and location available at GenBank by January 2018 were downloaded and, a final dataset of 747 DENV-1 sequences covering a total of 31 countries and spanning a period of 70 years (1977 to 2016) was analyzed, including the 30 sequences derived from this study. From this dataset, 171 sequences are from Brazil and are distributed according the geographic regions as follows: Midwest (n = 27), Southeast (n = 62), South (n = 1), Northeast (n = 38) and North (n = 42) and one with no information (Table S2). GenBank accession numbers, countries of origin and year of isolation of all included sequences are shown in Table S1. Nucleotide sequences were aligned using MAFFT v6.902b program and the alignments may be available from the authors upon request.

### DENV-1 Genotype V phylogenetic analysis

Phylogenetic relationships among genotype V sequences and sequences representing genotypes I, II, III and IV were resolved using a Maximum Likelihood (ML) tree inferred with PhyML^24^, under the GTR + I + Г4 model of nucleotide substitution as determined by automatic model selection by SMS: Smart Model Selecion in PhyML^25^ and the SPR branch-swapping heuristic tree search algorithm. A second phylogenetics analysis was performed in Sub-Clade I of genotype V, using a ML tree inferred with PhyML, under the TN93 + I + Г4 model of nucleotide substitution as determined by automatic model selection by SMS: Smart Model Selecion in PhyML. The reliability of the phylogenies was estimated with the approximate likelihood-ratio (aLRT) SH-like test^26^ and trees were visualized with FigTree v1.4.2 program^27^.

### Spatiotemporal dispersion of current lineages of DENV-1 genotype V in Brazil

The rate of nucleotide substitution per site per year (subs./site/year), the time to the most recent common ancestor (T_MRCA_) and the spatial diffusion were jointly estimated for the DENV-1 genotype V lineages using the Bayesian Markov Chain Monte Carlo (MCMC) statistical framework implemented in the BEAST v1.8 package^28^ with BEAGLE^29^ to improve run performance. The DENV-1 genotype V sequences were subdivided into three subsets that contained Brazilian sequences until 2016: Sub-Clade Ia (n = 106), Sub-Clade Ib (n = 56) and Clade II (n = 116) (Table S2). The spatiotemporal scale of evolutionary process for analysis was directly estimated from the sampling dates of the sequences using the GTR + Г4 (Sub-Clade Ia), TN93 + Г4 (Sub-Clade Ib) and TN93 + I + Г4 (Clade II) nucleotide substitution model as determined by jModelTest program^30^, a relaxed uncorrelated lognormal molecular clock model^31^, a Bayesian Skyline coalescent tree prior^32^ and reversible discrete phylogeography model^33^. MCMC was run sufficiently long to ensure stationary and convergence of parameters was assessed by calculating the Effective Sample Size (ESS) using TRACER v1.6 (http://tree.bio.ed.ac.uk/software/tracer/). Maximum clade credibility (MCC) trees were summarized using TreeAnnotator v1.8 and visualized with FigTree v1.4.2.

## Electronic supplementary material

Supplementary information

## Data Availability

All data generated during this study are included in this published article. The alignments may be available from the authors upon request.
