# Supplementary material for: DENV-1 Genotype V in Brazil: Spatiotemporal dispersion pattern reveals continuous co-circulation of distinct lineages until 2016
Source: Sci Rep. 2018 Nov 21;8:17160. doi: 10.1038/s41598-018-35622-x (PMC6249214; doi:10.1038/s41598-018-35622-x)
Supplement: Supplementary file 1 — Supplementary information [file 41598_2018_35622_MOESM1_ESM.pdf]

DENV-1 Genotype V in Brazil: Spatiotemporal dispersion pattern reveals continuous co-circulation of distinct lineages until 2016

Fernanda de Bruycker-Nogueira[1], Thiara Manuele Alves Souza[1], Thaís Chouin-Carneiro[1], Nieli Rodrigues da Costa Faria[2], Jaqueline Bastos Santos[2], Maria Celeste Torres[2], Izabel Letícia Cavalcanti Ramalho[3], Shirlei Ferreira de Aguiar[4], Rita Maria Ribeiro Nogueira[2], Ana Maria Bispo de Filippis[2], Flavia Barreto dos Santos[1,*]

[1]Viral Immunology Laboratory, Oswaldo Cruz Institute - FIOCRUZ, Rio de Janeiro, Brazil.

[2]Flavivirus Laboratory, Oswaldo Cruz Institute - FIOCRUZ, Rio de Janeiro, Brazil.

[3]Central Laboratory of Public Health of Ceará - LACEN-CE.

[4]Central Laboratory of Public Health of Rio de Janeiro - LACEN-RJ.

Correspondence and requests for materials should be addressed to FBN (nandanog@ioc.fiocruz.br) and FBS (flaviab@ioc.fiocruz.br)

Supplementary information

Table S1. DENV-1 Genotype V (clade cosmopolitan) dataset.

	ID	Genbank	Country	Year
1.	AF514889/AR/2000	AF514889	Argentina	2000
2.	AY206457/AR/2000	AY206457	Argentina	2000
3.	AF514885/AR/2000	AF514885	Argentina	2000
4.	AF514876/AR/2000	AF514876	Argentina	2000
5.	KC692510/AR/2009	KC692510	Argentina	2009
6.	KC692497/AR/2009	KC692497	Argentina	2009
7.	KC692502/AR/2009	KC692502	Argentina	2009
8.	KC692509/AR/2009	KC692509	Argentina	2009
9.	KC692506/AR/2009	KC692506	Argentina	2009
10.	KC692501/AR/2009	KC692501	Argentina	2009
11.	KC692508/AR/2009	KC692508	Argentina	2009
12.	KC692499/AR/2009	KC692499	Argentina	2009

13.	KC692500/AR/2009	KC692500	Argentina	2009
14.	KC692503/AR/2009	KC692503	Argentina	2009
15.	KC692495/AR/2009	KC692495	Argentina	2009
16.	KC692496/AR/2009	KC692496	Argentina	2009
17.	KC692505/AR/2009	KC692505	Argentina	2009
18.	KC692504/AR/2009	KC692504	Argentina	2009
19.	KC692507/AR/2009	KC692507	Argentina	2009
20.	KC692498/AR/2009	KC692498	Argentina	2009
21.	KC692511/AR/2010	KC692511	Argentina	2010
22.	KC692513/AR/2010	KC692513	Argentina	2010
23.	KC692517/AR/2010	KC692517	Argentina	2010
24.	KC692516/AR/2010	KC692516	Argentina	2010
25.	KC692514/AR/2010	KC692514	Argentina	2010
26.	KC692512/AR/2010	KC692512	Argentina	2010
27.	KC692515/AR/2010	KC692515	Argentina	2010
28.	KX768341/AR/2016	KX768341	Argentina	2016
29.	KX768342/AR/2016	KX768342	Argentina	2016
30.	KX768347/AR/2016	KX768347	Argentina	2016
31.	KX768348/AR/2016	KX768348	Argentina	2016
32.	KX768346/AR/2016	KX768346	Argentina	2016
33.	KX768343/AR/2016	KX768343	Argentina	2016
34.	KX768349/AR/2016	KX768349	Argentina	2016
35.	Y283850/AR/2016	Y283850	Argentina	2016
36.	KX768358/AR/2016	KX768358	Argentina	2016
37.	KX768351/AR/2016	KX768351	Argentina	2016
38.	KX768345/AR/2016	KX768345	Argentina	2016
39.	KX768354/AR/2016	KX768354	Argentina	2016
40.	KX768353/AR/2016	KX768353	Argentina	2016
41.	KX768352/AR/2016	KX768352	Argentina	2016
42.	KX768359/AR/2016	KX768359	Argentina	2016
43.	Y283848/AR/2016	Y283848	Argentina	2016
44.	KX768344/AR/2016	KX768344	Argentina	2016
45.	KX768350/AR/2016	KX768350	Argentina	2016
46.	KX768356/AR/2016	KX768356	Argentina	2016
47.	KX768357/AR/2016	KX768357	Argentina	2016
48.	Y283851/AR/2016	Y283851	Argentina	2016
49.	KX768355/AR/2016	KX768355	Argentina	2016
50.	KX768362/AR/2016	KX768362	Argentina	2016
51.	KX768360/AR/2016	KX768360	Argentina	2016
52.	KX768361/AR/2016	KX768361	Argentina	2016
53.	KX768339/AR/2016	KX768339	Argentina	2016
54.	Y283852/AR/2016	Y283852	Argentina	2016
55.	KX768340/AR/2016	KX768340	Argentina	2016
56.	KX768338/AR/2016	KX768338	Argentina	2016
57.	KX768364/AR/2016	KX768364	Argentina	2016
58.	KX768366/AR/2016	KX768366	Argentina	2016
59.	KX768363/AR/2016	KX768363	Argentina	2016
60.	KX768365/AR/2016	KX768365	Argentina	2016
61.	KX768375/AR/2016	KX768375	Argentina	2016
62.	KX768376/AR/2016	KX768376	Argentina	2016
63.	KX768370/AR/2016	KX768370	Argentina	2016
64.	KX768367/AR/2016	KX768367	Argentina	2016
65.	KY283849/AR/2016	KY283849	Argentina	2016
66.	KX768372/AR/2016	KX768372	Argentina	2016
67.	KX768369/AR/2016	KX768369	Argentina	2016
68.	KX768368/AR/2016	KX768368	Argentina	2016
69.	KX768373/AR/2016	KX768373	Argentina	2016
70.	KX768374/AR/2016	KX768374	Argentina	2016
71.	KX768371/AR/2016	KX768371	Argentina	2016
72.	KX768377/AR/2016	KX768377	Argentina	2016

73.	KX768380/AR/2016	KX768380	Argentina	2016
74.	KX768385/AR/2016	KX768385	Argentina	2016
75.	KX768384/AR/2016	KX768384	Argentina	2016
76.	KX768382/AR/2016	KX768382	Argentina	2016
77.	KX768381/AR/2016	KX768381	Argentina	2016
78.	KY283853/AR/2016	KY283853	Argentina	2016
79.	KX768383/AR/2016	KX768383	Argentina	2016
80.	KX768408/AR/2016	KX768408	Argentina	2016
81.	KX768410/AR/2016	KX768410	Argentina	2016
82.	KX768409/AR/2016	KX768409	Argentina	2016
83.	KX768378/AR/2016	KX768378	Argentina	2016
84.	KX768416/AR/2016	KX768416	Argentina	2016
85.	KX768407/AR/2016	KX768407	Argentina	2016
86.	KX768417/AR/2016	KX768417	Argentina	2016
87.	KX768399/AR/2016	KX768399	Argentina	2016
88.	KX768402/AR/2016	KX768402	Argentina	2016
89.	KX768412/AR/2016	KX768412	Argentina	2016
90.	KX768415/AR/2016	KX768415	Argentina	2016
91.	KX768419/AR/2016	KX768419	Argentina	2016
92.	KX768406/AR/2016	KX768406	Argentina	2016
93.	KX768413/AR/2016	KX768413	Argentina	2016
94.	KX768400/AR/2016	KX768400	Argentina	2016
95.	KX768404/AR/2016	KX768404	Argentina	2016
96.	KX768405/AR/2016	KX768405	Argentina	2016
97.	KX768396/AR/2016	KX768396	Argentina	2016
98.	KX768418/AR/2016	KX768418	Argentina	2016
99.	KX768389/AR/2016	KX768389	Argentina	2016
100.	KX768379/AR/2016	KX768379	Argentina	2016
101.	KX768390/AR/2016	KX768390	Argentina	2016
102.	KX768403/AR/2016	KX768403	Argentina	2016
103.	KX768391/AR/2016	KX768391	Argentina	2016
104.	KX768411/AR/2016	KX768411	Argentina	2016
105.	KX768388/AR/2016	KX768388	Argentina	2016
106.	KX768414/AR/2016	KX768414	Argentina	2016
107.	KX768398/AR/2016	KX768398	Argentina	2016
108.	KX768401/AR/2016	KX768401	Argentina	2016
109.	KX768387/AR/2016	KX768387	Argentina	2016
110.	KX768397/AR/2016	KX768397	Argentina	2016
111.	KX768393/AR/2016	KX768393	Argentina	2016
112.	KX768392/AR/2016	KX768392	Argentina	2016
113.	KX768394/AR/2016	KX768394	Argentina	2016
114.	KX768395/AR/2016	KX768395	Argentina	2016
115.	KX768386/AR/2016	KX768386	Argentina	2016
116.	AF425609/AW/1985	AF425609	Aruba	1985
117.	JN379473/AW/2004	JN379473	Aruba	2004
118.	JN379475/BS/1977	JN379475	Bahamas	1977
119.	JN379486/BB/1995	JN379486	Barbados	1995
120.	JN379487/BB/1999	JN379487	Barbados	1999
121.	JN379470/BB/2001	JN379470	Barbados	2001
122.	JN379471/BB/2001	JN379471	Barbados	2001
123.	JN379472/BB/2003	JN379472	Barbados	2003
124.	JN379474/BZ/2005	JN379474	Belize	2005
125.	AF425613/BRN/1982	AF425613	Brazil	1982
126.	JN122280/BRSE/RJ/1986	JN122280	Brazil	1986
127.	HQ026760/BRSE/RJ/1986	HQ026760	Brazil	1986
128.	KF672761/BRSE/BR/RJ/1988	KF672761	Brazil	1988
129.	KF672762/BRSE/BR/RJ/1989	KF672762	Brazil	1989
130.	AF226685/BRSE/RJ/1990	AF226685	Brazil	1990
131.	KF672791/BRCO/BR/MS/1991	KF672791	Brazil	1991

132.	HM450079/BRNE/BR/CE/1994	HM450079	Brazil	1994
133.	JX669467/BRNE/PE/1996	JX669467	Brazil	1996
134.	HM450080/BRN/BR/PA/1996	HM450080	Brazil	1996
135.	KF672770/BRSE/BR/MG/1997	KF672770	Brazil	1997
136.	KF672771/BRSE/BR/RJ/1997	KF672771	Brazil	1997
137.	KF672769/BRSE/BR/MG/1997	KF672769	Brazil	1997
138.	KF672773/BRNE/BR/RN/1997	KF672773	Brazil	1997
139.	KF672772/BRSE/BR/RJ/1997	KF672772	Brazil	1997
140.	AF311957/BRNE/PE/1997	AF311957	Brazil	1997
141.	AF311958/BRNE/PE/1997	AF311958	Brazil	1997
142.	JX669468/BRNE/PE/1997	JX669468	Brazil	1997
143.	AF311956/BRNE/PE/1997	AF311956	Brazil	1997
144.	JX669469/BRNE/PE/1997	JX669469	Brazil	1997
145.	HM450081/BRSE/BR/MG/1997	HM450081	Brazil	1997
146.	HM450082/BRN/BR/PA/1997	HM450082	Brazil	1997
147.	KF672776/BRSE/BR/MG/1998	KF672776	Brazil	1998
148.	KF672779/BRSE/BR/ES/1998	KF672779	Brazil	1998
149.	KF672775/BRNE/BR/CE/1998	KF672775	Brazil	1998
150.	KF672778/BRSE/BR/ES/1998	KF672778	Brazil	1998
151.	JX669470/BRNE/PE/1998	JX669470	Brazil	1998
152.	KF672774/BRSE/BR/RJ/1998	KF672774	Brazil	1998
153.	KF672777/BRSE/BR/MG/1998	KF672777	Brazil	1998
154.	HM450083/BRNE/BR/MA/1999	HM450083	Brazil	1999
155.	JX669471/BRNE/PE/1999	JX669471	Brazil	1999
156.	KF672792/BRSE/BR/RJ/1999	KF672792	Brazil	1999
157.	KF672781/BRSE/BR/RJ/1999	KF672781	Brazil	1999
158.	KF672780/BRSE/BR/RJ/1999	KF672780	Brazil	1999
159.	FJ850070/BRN/BR/2000	FJ850070	Brazil	2000
160.	FJ850071/BRN/BR/2000	FJ850071	Brazil	2000
161.	HM450087/BRNE/BR/CE/2000	HM450087	Brazil	2000
162.	HM450086/BRNE/BR/CE/2000	HM450086	Brazil	2000
163.	AY277665/BR/2000	AY277665	Brazil	2000
164.	KF672763/BRSE/BR/ES/2000	KF672763	Brazil	2000
165.	KF672782/BRSE/BR/RJ/2000	KF672782	Brazil	2000
166.	JX669472/BRNE/PE/2000	JX669472	Brazil	2000
167.	KF672787/BRNE/BR/PI/2000	KF672787	Brazil	2000
168.	HM450085/BRN/BR/AC/2000	HM450085	Brazil	2000
169.	HM450084/BRCO/BR/MT/2000	HM450084	Brazil	2000
170.	KF672789/BRSE/BR/ES/2001	KF672789	Brazil	2001
171.	HM450089/BRN/BR/RR/2001	HM450089	Brazil	2001
172.	HM450088/BRN/BR/AP/2001	HM450088	Brazil	2001
173.	KF672788/BRSE/BR/RJ/2001	KF672788	Brazil	2001
174.	KF672764/BRSE/BR/RJ/2001	KF672764	Brazil	2001
175.	HM450098/BRNE/BR/RN/2001	HM450098	Brazil	2001
176.	FJ850073/BRNE/BR/2001	FJ850073	Brazil	2001
177.	JX669474/BRNE/PE/2001	JX669474	Brazil	2001
178.	AF513110/BRS/PR/2001	AF513110	Brazil	2001
179.	JX669473/BRNE/PE/2001	JX669473	Brazil	2001
180.	FJ384655/BRCO/DF/2001	FJ384655	Brazil	2001
181.	AB519681/BRCO/BR/DF/2001	AB519681	Brazil	2001
182.	HM450094/BRN/BR/TO/2002	HM450094	Brazil	2002
183.	FJ850075/BRN/BR/2002	FJ850075	Brazil	2002

184.	HM450093/BRNE/BR/PI/2002	HM450093	Brazil	2002
185.	HM450105/BRN/BR/AM/2002	HM450105	Brazil	2002
186.	HM450095/BRN/BR/AM/2002	HM450095	Brazil	2002
187.	HM450092/BRNE/BR/PI/2002	HM450092	Brazil	2002
188.	JX669475/BRNE/PE/2002	JX669475	Brazil	2002
189.	KF672783/BRSE/BR/ES/2002	KF672783	Brazil	2002
190.	HM450091/BRCO/BR/MT/2002	HM450091	Brazil	2002
191.	HM450090/BRCO/BR/MT/2002	HM450090	Brazil	2002
192.	FJ850077/BRN/BR/2003	FJ850077	Brazil	2003
193.	HM450096/BRNE/BR/MA/2003	HM450096	Brazil	2003
194.	HM450097/BRN/BR/PA/2004	HM450097	Brazil	2004
195.	FJ850081/BRN/BR/2004	FJ850081	Brazil	2004
196.	HM450099/BRN/BR/AP/2005	HM450099	Brazil	2005
197.	FJ850084/BRN/BR/2005	FJ850084	Brazil	2005
198.	FJ850087/BRN/BR/2006	FJ850087	Brazil	2006
199.	HM450100/BRN/BR/PA/2006	HM450100	Brazil	2006
200.	FJ850090/BRN/BR/2007	FJ850090	Brazil	2007
201.	HM450101/BRN/BR/PA/2007	HM450101	Brazil	2007
202.	HM450077/BRN/BR/PA/2007	HM450077	Brazil	2007
203.	HM450078/BRN/BR/PA/2007	HM450078	Brazil	2007
204.	HM450102/BRN/BR/RR/2007	HM450102	Brazil	2007
205.	HM450103/BRN/BR/AM/2007	HM450103	Brazil	2007
206.	GU131863/BRSE/BR/SP/2008	GU131863	Brazil	2008
207.	KF444789/BRN/BR/RR/2008	KF444789	Brazil	2008
208.	FJ850093/BRN/BR/2008	FJ850093	Brazil	2008
209.	HM450104/BRN/BR/RR/2008	HM450104	Brazil	2008
210.	HM043709/BRSE/BR/ES/2009	HM043709	Brazil	2009
211.	HQ026761/BRSE/BR/RJ/2009	HQ026761	Brazil	2009
212.	HM043710/BRSE/BR/RJ/2009	HM043710	Brazil	2009
213.	KF444783/BRN/BR/RR/2009	KF444783	Brazil	2009
214.	KF444782/BRN/BR/RR/2009	KF444782	Brazil	2009
215.	KF444791/BRN/BR/RR/2009	KF444791	Brazil	2009
216.	KF444781/BRN/BR/RR/2009	KF444781	Brazil	2009
217.	KF444784/BRN/BR/RR/2009	KF444784	Brazil	2009
218.	KF444785/BRN/BR/RR/2009	KF444785	Brazil	2009
219.	KF444780/BRN/BR/RR/2009	KF444780	Brazil	2009
220.	KF444790/BRN/BR/RR/2009	KF444790	Brazil	2009
221.	JX669464/BRNE/BR/2010	JX669464	Brazil	2010
222.	JX669461/BRNE/BR/2010	JX669461	Brazil	2010
223.	JX669465/BRNE/BR/2010	JX669465	Brazil	2010
224.	JN982362/BRNE/BR/CE/2010	JN982362	Brazil	2010
225.	KF672759/BRSE/BR/RJ/2010	KF672759	Brazil	2010
226.	HQ026762/BRSE/BR/RJ/2010	HQ026762	Brazil	2010
227.	HQ696614/BRSE/BR/RJ/2010	HQ696614	Brazil	2010
228.	HQ696613/BRSE/BR/RJ/2010	HQ696613	Brazil	2010
229.	KF719187/BRSE/BR/ES/2010	KF719187	Brazil	2010
230.	KF672785/BRSE/BR/ES/2010	KF672785	Brazil	2010
231.	KF444787/BRN/BR/RR/2010	KF444787	Brazil	2010
232.	KF444788/BRN/BR/RR/2010	KF444788	Brazil	2010
233.	KF444786/BRN/BR/RR/2010	KF444786	Brazil	2010
234.	JN713897/BRN/BR/RR/2010	JN713897	Brazil	2010
235.	KF444792/BRN/BR/RR/2010	KF444792	Brazil	2010

236.	JQ015185/BRNE/BR/AL/2010	JQ015185	Brazil	2010
237.	JQ015184/BRNE/BR/AL/2010	JQ015184	Brazil	2010
238.	JX669463/BRNE/PE/2010	JX669463	Brazil	2010
239.	JX669466/BRNE/PE/2010	JX669466	Brazil	2010
240.	JX669462/BRNE/PE/2010	JX669462	Brazil	2010
241.	HQ696612/BRCO/MS/2010	HQ696612	Brazil	2010
242.	KF672768/BRCO/BR/MS/2010	KF672768	Brazil	2010
243.	KF672786/BRSE/BR/RJ/2010	KF672786	Brazil	2010
244.	KF672784/BRSE/BR/RJ/2011	KF672784	Brazil	2011
245.	KF672767/BRSE/BR/RJ/2011	KF672767	Brazil	2011
246.	KF672765/BRSE/BR/RJ/2011	KF672765	Brazil	2011
247.	KF672760/BRSE/BR/RJ/2011	KF672760	Brazil	2011
248.	JN122281/BRSE/RJ/2011	JN122281	Brazil	2011
249.	KF672790/BRSE/BR/RJ/2011	KF672790	Brazil	2011
250.	KF672766/BRSE/BR/RJ/2011	KF672766	Brazil	2011
251.	2071/BRSE/BR/RJ/2012	MH401971	Brazil	2012
252.	3599/BRSE/BR/RJ/2012	MH311981	Brazil	2012
253.	2612/BRSE/BR/RJ/2012	2612/BRS	Brazil	2012
254.	Lac1/BRSE/BR/RJ/2012	MH401990	Brazil	2012
255.	3239/BRSE/BR/RJ/2012	MH401973	Brazil	2012
256.	3246/BRSE/BR/RJ/2012	MH401974	Brazil	2012
257.	Lac16/BRSE/BR/RJ/2012	MH401992	Brazil	2012
258.	Lac5/BRSE/BR/RJ/2012	MH401991	Brazil	2012
259.	7436/BRCO/BR/MS/2013	MH401976	Brazil	2013
260.	7276/BRCO/BR/MS/2013	MH401975	Brazil	2013
261.	92/BRSE/BR/RJ/2013	MH401977	Brazil	2013
262.	KP858112/BRCO/BR/GO/2013	KP858112	Brazil	2013
263.	KP858113/BRCO/BR/GO/2013	KP858113	Brazil	2013
264.	KP858117/BRCO/BR/GO/2013	KP858117	Brazil	2013
265.	KP858109/BRCO/BR/GO/2013	KP858109	Brazil	2013
266.	KP858115/BRCO/BR/GO/2013	KP858115	Brazil	2013
267.	KP858111/BRCO/BR/GO/2013	KP858111	Brazil	2013
268.	KP858118/BRCO/BR/GO/2013	KP858118	Brazil	2013
269.	KP858110/BRCO/BR/GO/2013	KP858110	Brazil	2013
270.	KP858105/BRCO/BR/GO/2013	KP858105	Brazil	2013
271.	KP858106/BRCO/BR/GO/2013	KP858106	Brazil	2013
272.	KP858108/BRCO/BR/GO/2013	KP858108	Brazil	2013
273.	KP858116/BRCO/BR/GO/2013	KP858116	Brazil	2013
274.	KP858119/BRCO/BR/GO/2013	KP858119	Brazil	2013
275.	KP858114/BRCO/BR/GO/2013	KP858114	Brazil	2013
276.	KP858107/BRCO/BR/GO/2013	KP858107	Brazil	2013
277.	283/BRNE/BR/CE/2014	MH401987	Brazil	2014
278.	288/BRNE/BR/CE/2014	MH401988	Brazil	2014
279.	478/BRSE/BR/RJ/2014	MH401979	Brazil	2014
280.	494/BRNE/BR/SE/2014	MH401980	Brazil	2014
281.	297/BRNE/BR/CE/2014	297/BRNE	Brazil	2014
282.	05/BRSE/BR/RJ/2014	MH401978	Brazil	2014
283.	2072/BRSE/BR/RJ/2015	MH401984	Brazil	2015
284.	3171/BRSE/BR/RJ/2015	MH401985	Brazil	2015
285.	134/BRSE/BR/RJ/2015	MH401982	Brazil	2015
286.	30/BRSE/BR/RJ/2015	MH401981	Brazil	2015
287.	738/BRSE/BR/RJ/2015	MH401983	Brazil	2015

288.	67/BRN/BR/AP/2015	67/BRN/B	Brazil	2015
289.	148/BRNE/BR/SE/2015	MH401986	Brazil	2015
290.	Lac7/BRSE/BR/RJ/2015	MH401993	Brazil	2015
291.	Lac9/BRSE/BR/RJ/2016	MH401996	Brazil	2016
292.	KCMM25/BRCO/BR/MS/2016	MH401998	Brazil	2016
293.	Lac4/BRSE/BR/RJ/2016	MH401994	Brazil	2016
294.	Lac8/BRSE/BR/RJ/2016	MH401995	Brazil	2016
295.	VAOR28/BRCO/BR/MS/2016	MH401999	Brazil	2016
296.	GQ868601/VG/1985	GQ868601	British Virgin Islands	1985
297.	JF804023/VG/1987	JF804023	British Virgin Islands	1987
298.	KX372687/CN/2016	KX372687	China	2016
299.	AF425616/CO/1985	AF425616	Colombia	1985
300.	AF425617/CO/1996	AF425617	Colombia	1996
301.	KJ189302/CO/1998	KJ189302	Colombia	1998
302.	GQ868560/CO/1998	GQ868560	Colombia	1998
303.	GQ868559/CO/1998	GQ868559	Colombia	1998
304.	KJ189303/CO/1998	KJ189303	Colombia	1998
305.	GQ868561/CO/1999	GQ868561	Colombia	1999
306.	GU131948/CO/2001	GU131948	Colombia	2001
307.	KJ189304/CO/2005	KJ189304	Colombia	2005
308.	GQ868562/CO/2005	GQ868562	Colombia	2005
309.	GQ868564/CO/2006	GQ868564	Colombia	2006
310.	GQ868563/CO/2006	GQ868563	Colombia	2006
311.	GQ868565/CO/2006	GQ868565	Colombia	2006
312.	GU131949/CO/2006	GU131949	Colombia	2006
313.	GQ868568/CO/2007	GQ868568	Colombia	2007
314.	GQ868567/CO/2007	GQ868567	Colombia	2007
315.	GQ868569/CO/2007	GQ868569	Colombia	2007
316.	GQ868566/CO/2007	GQ868566	Colombia	2007
317.	JF804015/CO/2007	JF804015	Colombia	2007
318.	GQ868570/CO/2008	GQ868570	Colombia	2008
319.	AY153755/CR/1993	AY153755	Costa Rica	1993
320.	AY153756/CR/1993	AY153756	Costa Rica	1993
321.	AY153757/CR/1993	AY153757	Costa Rica	1993
322.	JF804016/CR/2005	JF804016	Costa Rica	2005
323.	JF804017/DO/2007	JF804017	Dominican Republic	2007
324.	JN819417/SV/1993	JN819417	El Salvador	1993
325.	EU448414/SV/2006	EU448414	El Salvador	2006
326.	JX891659/SV/2012	JX891659	El Salvador	2012
327.	JX891661/SV/2012	JX891661	El Salvador	2012
328.	JX891660/SV/2012	JX891660	El Salvador	2012
329.	AF226687/GF/1989	AF226687	French Guiana	1989
330.	JN379480/GD/1977	JN379480	Granada	1977
331.	AF425618/GD/1977	AF425618	Granada	1977
332.	JN379476/GD/1977	JN379476	Granada	1977
333.	JN379479/GD/1977	JN379479	Granada	1977
334.	JN379478/GD/1977	JN379478	Granada	1977
335.	JN379477/GD/1977	JN379477	Granada	1977
336.	JN379482/GD/1978	JN379482	Granada	1978
337.	JN379481/GD/1978	JN379481	Granada	1978
338.	JN379484/GD/1981	JN379484	Granada	1981
339.	JN415506/GY/2008	JN415506	Guyana	2008
340.	JF969282/HT/2010	JF969282	Haiti	2010
341.	JF969280/HT/2010	JF969280	Haiti	2010
342.	JF969281/HT/2010	JF969281	Haiti	2010
343.	JF969283/HT/2010	JF969283	Haiti	2010
344.	JF969284/HT/2010	JF969284	Haiti	2010
345.	JF967804/HN/2008	JF967804	Honduras	2008
346.	JF297572/IN/1962	JF297572	India	1962

347.	JF297573/IN/1962	JF297573	India	1962
348.	JF297576/IN/1963	JF297576	India	1963
349.	JQ922544/IN/1963	JQ922544	India	1963
350.	JF297577/IN/1963	JF297577	India	1963
351.	JF297574/IN/1963	JF297574	India	1963
352.	JF297575/IN/1963	JF297575	India	1963
353.	DQ016653/IN/2003	DQ016653	India	2003
354.	DQ016654/IN/2003	DQ016654	India	2003
355.	DQ016655/IN/2003	DQ016655	India	2003
356.	JF297583/IN/2005	JF297583	India	2005
357.	EU448413/IN/2006	EU448413	India	2006
358.	JN903578/IN/2007	JN903578	India	2007
359.	AF425621/JM/1977	AF425621	Jamaica	1977
360.	JF804018/MQ/1989	JF804018	Martinique	1989
361.	JN022597/MQ/2008	JN022597	Martinique	2008
362.	JN022598/MQ/2008	JN022598	Martinique	2008
363.	JN022599/MQ/2008	JN022599	Martinique	2008
364.	AF425623/MX/1980	AF425623	Mexico	1980
365.	DQ341188/MX/1982	DQ341188	Mexico	1982
366.	AF425624/MX/1983	AF425624	Mexico	1983
367.	DQ341189/MX/1984	DQ341189	Mexico	1984
368.	DQ341190/MX/1984	DQ341190	Mexico	1984
369.	DQ341191/MX/1986	DQ341191	Mexico	1986
370.	DQ341192/MX/1994	DQ341192	Mexico	1994
371.	DQ341193/MX/1995	DQ341193	Mexico	1995
372.	DQ341194/MX/1995	DQ341194	Mexico	1995
373.	GU131958/MX/2006	GU131958	Mexico	2006
374.	GQ868499/MX/2006	GQ868499	Mexico	2006
375.	KF955415/MX/2006	KF955415	Mexico	2006
376.	HM171568/MX/2006	HM171568	Mexico	2006
377.	HM171570/MX/2006	HM171570	Mexico	2006
378.	KF955417/MX/2006	KF955417	Mexico	2006
379.	HM171562/MX/2006	HM171562	Mexico	2006
380.	HM171567/MX/2006	HM171567	Mexico	2006
381.	HM171566/MX/2006	HM171566	Mexico	2006
382.	HM171559/MX/2006	HM171559	Mexico	2006
383.	HM171557/MX/2006	HM171557	Mexico	2006
384.	HM171560/MX/2006	HM171560	Mexico	2006
385.	HM171558/MX/2006	HM171558	Mexico	2006
386.	GQ868498/MX/2006	GQ868498	Mexico	2006
387.	GU131960/MX/2007	GU131960	Mexico	2007
388.	KJ189321/MX/2007	KJ189321	Mexico	2007
389.	GU131966/MX/2007	GU131966	Mexico	2007
390.	HM171565/MX/2007	HM171565	Mexico	2007
391.	HM171564/MX/2007	HM171564	Mexico	2007
392.	HM171561/MX/2007	HM171561	Mexico	2007
393.	KJ189319/MX/2007	KJ189319	Mexico	2007
394.	GU131968/MX/2007	GU131968	Mexico	2007
395.	GQ868503/MX/2007	GQ868503	Mexico	2007
396.	KF955443/MX/2007	KF955443	Mexico	2007
397.	GQ868509/MX/2007	GQ868509	Mexico	2007
398.	KF955442/MX/2007	KF955442	Mexico	2007
399.	GU131961/MX/2007	GU131961	Mexico	2007
400.	GU131976/MX/2007	GU131976	Mexico	2007
401.	GU131964/MX/2007	GU131964	Mexico	2007
402.	KJ189318/MX/2007	KJ189318	Mexico	2007
403.	KF955428/MX/2007	KF955428	Mexico	2007
404.	GQ868517/MX/2007	GQ868517	Mexico	2007
405.	GQ868524/MX/2007	GQ868524	Mexico	2007
406.	GQ868510/MX/2007	GQ868510	Mexico	2007

407.	KJ189328/MX/2007	KJ189328	Mexico	2007
408.	GQ868505/MX/2007	GQ868505	Mexico	2007
409.	GQ868502/MX/2007	GQ868502	Mexico	2007
410.	KJ189326/MX/2007	KJ189326	Mexico	2007
411.	GQ868508/MX/2007	GQ868508	Mexico	2007
412.	GQ868520/MX/2007	GQ868520	Mexico	2007
413.	GU131979/MX/2007	GU131979	Mexico	2007
414.	GQ868522/MX/2007	GQ868522	Mexico	2007
415.	GQ868528/MX/2007	GQ868528	Mexico	2007
416.	GU131981/MX/2007	GU131981	Mexico	2007
417.	GQ868513/MX/2007	GQ868513	Mexico	2007
418.	KJ189317/MX/2007	KJ189317	Mexico	2007
419.	GQ868511/MX/2007	GQ868511	Mexico	2007
420.	HM171569/MX/2007	HM171569	Mexico	2007
421.	KJ189324/MX/2007	KJ189324	Mexico	2007
422.	KF955420/MX/2007	KF955420	Mexico	2007
423.	GU131973/MX/2007	GU131973	Mexico	2007
424.	GQ868521/MX/2007	GQ868521	Mexico	2007
425.	HQ166035/MX/2007	HQ166035	Mexico	2007
426.	KJ189330/MX/2007	KJ189330	Mexico	2007
427.	GU131980/MX/2007	GU131980	Mexico	2007
428.	GQ868523/MX/2007	GQ868523	Mexico	2007
429.	KJ189329/MX/2007	KJ189329	Mexico	2007
430.	HQ166036/MX/2007	HQ166036	Mexico	2007
431.	GQ868518/MX/2007	GQ868518	Mexico	2007
432.	KF955427/MX/2007	KF955427	Mexico	2007
433.	GU131965/MX/2007	GU131965	Mexico	2007
434.	KJ189323/MX/2007	KJ189323	Mexico	2007
435.	HM171563/MX/2007	HM171563	Mexico	2007
436.	GQ868536/MX/2008	GQ868536	Mexico	2008
437.	KJ189338/MX/2008	KJ189338	Mexico	2008
438.	GQ868535/MX/2008	GQ868535	Mexico	2008
439.	KJ189339/MX/2008	KJ189339	Mexico	2008
440.	KJ189313/MX/2008	KJ189313	Mexico	2008
441.	GQ868539/MX/2008	GQ868539	Mexico	2008
442.	KJ189333/MX/2008	KJ189333	Mexico	2008
443.	KJ189332/MX/2008	KJ189332	Mexico	2008
444.	KF955433/MX/2008	KF955433	Mexico	2008
445.	KJ189331/MX/2008	KJ189331	Mexico	2008
446.	KJ189337/MX/2008	KJ189337	Mexico	2008
447.	GQ868529/MX/2008	GQ868529	Mexico	2008
448.	GU131984/MX/2008	GU131984	Mexico	2008
449.	GU131983/MX/2008	GU131983	Mexico	2008
450.	KJ189315/MX/2008	KJ189315	Mexico	2008
451.	KJ189340/MX/2008	KJ189340	Mexico	2008
452.	KJ189336/MX/2008	KJ189336	Mexico	2008
453.	KJ189335/MX/2008	KJ189335	Mexico	2008
454.	GQ868534/MX/2008	GQ868534	Mexico	2008
455.	KJ189312/MX/2008	KJ189312	Mexico	2008
456.	GQ868533/MX/2008	GQ868533	Mexico	2008
457.	GQ868537/MX/2008	GQ868537	Mexico	2008
458.	KJ189345/MX/2009	KJ189345	Mexico	2009
459.	KJ189342/MX/2009	KJ189342	Mexico	2009
460.	KJ189316/MX/2009	KJ189316	Mexico	2009
461.	KJ189344/MX/2009	KJ189344	Mexico	2009
462.	KJ189346/MX/2009	KJ189346	Mexico	2009
463.	KJ189347/MX/2009	KJ189347	Mexico	2009
464.	JQ065957/MX/2010	JQ065957	Mexico	2010
465.	JQ065921/MX/2010	JQ065921	Mexico	2010
466.	JQ065917/MX/2010	JQ065917	Mexico	2010

467.	JQ065938/MX/2010	JQ065938	Mexico	2010
468.	JQ065907/MX/2010	JQ065907	Mexico	2010
469.	JQ065950/MX/2010	JQ065950	Mexico	2010
470.	JQ065903/MX/2010	JQ065903	Mexico	2010
471.	JQ065945/MX/2010	JQ065945	Mexico	2010
472.	JQ065942/MX/2010	JQ065942	Mexico	2010
473.	JQ920432/MX/2010	JQ920432	Mexico	2010
474.	JQ920429/MX/2010	JQ920429	Mexico	2010
475.	JQ065928/MX/2010	JQ065928	Mexico	2010
476.	JQ065922/MX/2010	JQ065922	Mexico	2010
477.	JQ065911/MX/2010	JQ065911	Mexico	2010
478.	JQ065951/MX/2010	JQ065951	Mexico	2010
479.	JQ065932/MX/2010	JQ065932	Mexico	2010
480.	JQ065947/MX/2010	JQ065947	Mexico	2010
481.	JQ065948/MX/2010	JQ065948	Mexico	2010
482.	JQ065934/MX/2010	JQ065934	Mexico	2010
483.	JQ920428/MX/2010	JQ920428	Mexico	2010
484.	JQ920430/MX/2010	JQ920430	Mexico	2010
485.	JQ065919/MX/2010	JQ065919	Mexico	2010
486.	JQ065954/MX/2010	JQ065954	Mexico	2010
487.	JQ065956/MX/2010	JQ065956	Mexico	2010
488.	JQ065924/MX/2010	JQ065924	Mexico	2010
489.	JQ065899/MX/2010	JQ065899	Mexico	2010
490.	JQ065930/MX/2010	JQ065930	Mexico	2010
491.	JQ065915/MX/2010	JQ065915	Mexico	2010
492.	JQ065949/MX/2010	JQ065949	Mexico	2010
493.	JQ065939/MX/2010	JQ065939	Mexico	2010
494.	JQ065925/MX/2010	JQ065925	Mexico	2010
495.	JQ065943/MX/2010	JQ065943	Mexico	2010
496.	JQ065935/MX/2010	JQ065935	Mexico	2010
497.	JQ920431/MX/2010	JQ920431	Mexico	2010
498.	JQ065941/MX/2010	JQ065941	Mexico	2010
499.	JQ065916/MX/2010	JQ065916	Mexico	2010
500.	JQ065914/MX/2010	JQ065914	Mexico	2010
501.	JQ065946/MX/2010	JQ065946	Mexico	2010
502.	KJ189349/MX/2011	KJ189349	Mexico	2011
503.	KJ189306/MX/2011	KJ189306	Mexico	2011
504.	KJ189307/MX/2011	KJ189307	Mexico	2011
505.	KM279414/MX/2011	KM279414	Mexico	2011
506.	KM279413/MX/2012	KM279413	Mexico	2012
507.	KM279416/MX/2012	KM279416	Mexico	2012
508.	KM279415/MX/2012	KM279415	Mexico	2012
509.	KM279419/MX/2012	KM279419	Mexico	2012
510.	KM279411/MX/2012	KM279411	Mexico	2012
511.	KM279412/MX/2012	KM279412	Mexico	2012
512.	KM279420/MX/2012	KM279420	Mexico	2012
513.	DQ016656/NI/1996	DQ016656	Nicaragua	1996
514.	DQ016657/NI/2003	DQ016657	Nicaragua	2003
515.	GQ199872/NI/2004	GQ199872	Nicaragua	2004
516.	FJ898437/NI/2004	FJ898437	Nicaragua	2004
517.	GQ199875/NI/2004	GQ199875	Nicaragua	2004
518.	GQ199873/NI/2004	GQ199873	Nicaragua	2004
519.	GQ199867/NI/2004	GQ199867	Nicaragua	2004
520.	EU596501/NI/2004	EU596501	Nicaragua	2004
521.	FJ024483/NI/2005	FJ024483	Nicaragua	2005
522.	FJ024482/NI/2005	FJ024482	Nicaragua	2005
523.	KF955403/NI/2005	KF955403	Nicaragua	2005
524.	FJ410290/NI/2005	FJ410290	Nicaragua	2005
525.	EU482617/NI/2005	EU482617	Nicaragua	2005
526.	EU596502/NI/2005	EU596502	Nicaragua	2005

527.	FJ024478/NI/2005	FJ024478	Nicaragua	2005
528.	EU482619/NI/2005	EU482619	Nicaragua	2005
529.	FJ547089/NI/2005	FJ547089	Nicaragua	2005
530.	FJ850114/NI/2005	FJ850114	Nicaragua	2005
531.	KF955409/NI/2005	KF955409	Nicaragua	2005
532.	FJ432721/NI/2005	FJ432721	Nicaragua	2005
533.	FJ432720/NI/2005	FJ432720	Nicaragua	2005
534.	FJ850113/NI/2005	FJ850113	Nicaragua	2005
535.	JN819402/NI/2005	JN819402	Nicaragua	2005
536.	EU482615/NI/2005	EU482615	Nicaragua	2005
537.	FJ024485/NI/2005	FJ024485	Nicaragua	2005
538.	FJ024480/NI/2005	FJ024480	Nicaragua	2005
539.	EU482618/NI/2005	EU482618	Nicaragua	2005
540.	FJ182002/NI/2005	FJ182002	Nicaragua	2005
541.	KF955404/NI/2005	KF955404	Nicaragua	2005
542.	EU596503/NI/2005	EU596503	Nicaragua	2005
543.	EU596504/NI/2005	EU596504	Nicaragua	2005
544.	FJ024481/NI/2005	FJ024481	Nicaragua	2005
545.	EU482616/NI/2005	EU482616	Nicaragua	2005
546.	FJ024484/NI/2005	FJ024484	Nicaragua	2005
547.	FJ873814/NI/2005	FJ873814	Nicaragua	2005
548.	FJ024423/NI/2005	FJ024423	Nicaragua	2005
549.	FJ562104/NI/2006	FJ562104	Nicaragua	2006
550.	JN819403/NI/2006	JN819403	Nicaragua	2006
551.	FJ024479/NI/2006	FJ024479	Nicaragua	2006
552.	FJ547068/NI/2006	FJ547068	Nicaragua	2006
553.	FJ810419/NI/2006	FJ810419	Nicaragua	2006
554.	FJ898433/NI/2007	FJ898433	Nicaragua	2007
555.	KF955410/NI/2008	KF955410	Nicaragua	2008
556.	GQ199859/NI/2008	GQ199859	Nicaragua	2008
557.	GQ199858/NI/2008	GQ199858	Nicaragua	2008
558.	GQ199857/NI/2008	GQ199857	Nicaragua	2008
559.	FJ547088/NI/2008	FJ547088	Nicaragua	2008
560.	JF937635/NI/2009	JF937635	Nicaragua	2009
561.	JQ287666/NI/2009	JQ287666	Nicaragua	2009
562.	JF937645/NI/2009	JF937645	Nicaragua	2009
563.	JF937644/NI/2009	JF937644	Nicaragua	2009
564.	AY277664/PY/1999	AY277664	Paraguay	1999
565.	AF514878/PY/2000	AF514878	Paraguay	2000
566.	AY277659/PY/2000	AY277659	Paraguay	2000
567.	AY277666/PY/2000	AY277666	Paraguay	2000
568.	AF514883/PY/2000	AF514883	Paraguay	2000
569.	AF425626/PE/1991	AF425626	Peru	1991
570.	FJ562106/PR/1986	FJ562106	Puerto Rico	1986
571.	FJ410190/PR/1987	FJ410190	Puerto Rico	1987
572.	FJ478458/PR/1987	FJ478458	Puerto Rico	1987
573.	FJ410187/PR/1992	FJ410187	Puerto Rico	1992
574.	FJ410186/PR/1992	FJ410186	Puerto Rico	1992
575.	FJ547087/PR/1992	FJ547087	Puerto Rico	1992
576.	FJ410184/PR/1993	FJ410184	Puerto Rico	1993
577.	FJ410183/PR/1993	FJ410183	Puerto Rico	1993
578.	FJ410185/PR/1993	FJ410185	Puerto Rico	1993
579.	FJ562105/PR/1993	FJ562105	Puerto Rico	1993
580.	FJ410175/PR/1994	FJ410175	Puerto Rico	1994
581.	FJ410179/PR/1994	FJ410179	Puerto Rico	1994
582.	FJ205875/PR/1995	FJ205875	Puerto Rico	1995
583.	FJ410181/PR/1995	FJ410181	Puerto Rico	1995
584.	KF955439/PR/1995	KF955439	Puerto Rico	1995
585.	FJ410180/PR/1995	FJ410180	Puerto Rico	1995
586.	FJ410174/PR/1995	FJ410174	Puerto Rico	1995

587.	FJ390374/PR/1995	FJ390374	Puerto Rico	1995
588.	FJ205874/PR/1995	FJ205874	Puerto Rico	1995
589.	FJ547086/PR/1995	FJ547086	Puerto Rico	1995
590.	KF921911/PR/1996	KF921911	Puerto Rico	1996
591.	FJ410182/PR/1996	FJ410182	Puerto Rico	1996
592.	FJ410189/PR/1996	FJ410189	Puerto Rico	1996
593.	FJ478457/PR/1996	FJ478457	Puerto Rico	1996
594.	FJ410188/PR/1996	FJ410188	Puerto Rico	1996
595.	JF804021/PR/1998	JF804021	Puerto Rico	1998
596.	KF955438/PR/1998	KF955438	Puerto Rico	1998
597.	EU482567/PR/1998	EU482567	Puerto Rico	1998
598.	FJ205873/PR/1998	FJ205873	Puerto Rico	1998
599.	FJ390380/PR/1998	FJ390380	Puerto Rico	1998
600.	KC812277/PR/1998	KC812277	Puerto Rico	1998
601.	EU482592/PR/1998	EU482592	Puerto Rico	1998
602.	KF955437/PR/1998	KF955437	Puerto Rico	1998
603.	FJ390378/PR/1998	FJ390378	Puerto Rico	1998
604.	FJ205872/PR/1998	FJ205872	Puerto Rico	1998
605.	FJ410173/PR/1998	FJ410173	Puerto Rico	1998
606.	FJ390379/PR/1998	FJ390379	Puerto Rico	1998
607.	EU482591/PR/2006	EU482591	Puerto Rico	2006
608.	JF804022/PR/2007	JF804022	Puerto Rico	2007
609.;	KJ189363/PR/2010	KJ189363	Puerto Rico	2010
610.	KJ189360/PR/2010	KJ189360	Puerto Rico	2010
611.	KJ189361/PR/2010	KJ189361	Puerto Rico	2010
612.	KJ189367/PR/2010	KJ189367	Puerto Rico	2010
613.	KJ189365/PR/2010	KJ189365	Puerto Rico	2010
614.	KJ189366/PR/2010	KJ189366	Puerto Rico	2010
615.	KJ189364/PR/2010	KJ189364	Puerto Rico	2010
616.	KJ189362/PR/2010	KJ189362	Puerto Rico	2010
617.	JX402207/PR/2010	JX402207	Puerto Rico	2010
618.	JX402211/PR/2010	JX402211	Puerto Rico	2010
619.	JX402209/PR/2010	JX402209	Puerto Rico	2010
620.	JX402208/PR/2010	JX402208	Puerto Rico	2010
621.	JX402210/PR/2010	JX402210	Puerto Rico	2010
622.	JX402212/PR/2010	JX402212	Puerto Rico	2010
623.	JX402213/PR/2010	JX402213	Puerto Rico	2010
624.	KJ189358/PR/2012	KJ189358	Puerto Rico	2012
625.	KJ189355/PR/2012	KJ189355	Puerto Rico	2012
626.	KJ189352/PR/2012	KJ189352	Puerto Rico	2012
627.	KJ189357/PR/2012	KJ189357	Puerto Rico	2012
628.	KJ189356/PR/2012	KJ189356	Puerto Rico	2012
629.	KJ189353/PR/2012	KJ189353	Puerto Rico	2012
630.	KJ189350/PR/2012	KJ189350	Puerto Rico	2012
631.	KJ189354/PR/2012	KJ189354	Puerto Rico	2012
632.	KJ189359/PR/2012	KJ189359	Puerto Rico	2012
633.	KJ189351/PR/2012	KJ189351	Puerto Rico	2012
634.	DQ285554/RE/2004	DQ285554	Reunion Island	2004
635.	DQ285559/RE/2004	DQ285559	Reunion Island	2004
636.	KY829115/SB/2016	KY829115	Saint Barthelemy	2016
637.	M87512/SG/1990	M87512	Singapore	1990
638.	EU081258/SG/2005	EU081258	Singapore	2005
639.	JN379485/SR/1981	JN379485	Suriname	1981
640.	AF425631/TT/1978	AF425631	Trinidad and Tobago	1978
641.	JN379483/TT/1981	JN379483	Trinidad and Tobago	1981
642.	AF425639/TT/1986	AF425639	Trinidad and Tobago	1986
643.	JQ425067/US/2009	JQ425067	United States	2009
644.	JQ425061/US/2009	JQ425061	United States	2009
645.	JQ425063/US/2009	JQ425063	United States	2009
646.	JQ425068/US/2009	JQ425068	United States	2009

647.	JQ425062/US/2009	JQ425062	United States	2009
648.	JQ425064/US/2009	JQ425064	United States	2009
649.	JQ425066/US/2009	JQ425066	United States	2009
650.	JQ425065/US/2009	JQ425065	United States	2009
651.	JQ045563/US/2010	JQ045563	United States	2010
652.	JQ045562/US/2010	JQ045562	United States	2010
653.	JQ045561/US/2010	JQ045561	United States	2010
654.	JQ675358/US/2010	JQ675358	United States	2010
655.	JQ045564/US/2010	JQ045564	United States	2010
656.	JF519855/US/2010	JF519855	United States	2010
657.	JQ425072/US/2010	JQ425072	United States	2010
658.	JQ425071/US/2010	JQ425071	United States	2010
659.	JQ425070/US/2010	JQ425070	United States	2010
660.	JQ425069/US/2010	JQ425069	United States	2010
661.	KJ415284/US/2013	KJ415284	United States	2013
662.	KM458188/US/2014	KM458188	United States	2014
663.	KM458186/US/2014	KM458186	United States	2014
664.	KM458189/US/2014	KM458189	United States	2014
665.	KM458187/US/2014	KM458187	United States	2014
666.	KM458190/US/2014	KM458190	United States	2014
667.	AF425636/VE/1994	AF425636	Venezuela	1994
668.	AF425637/VE/1994	AF425637	Venezuela	1994
669.	AF425632/VE/1995	AF425632	Venezuela	1995
670.	AF425638/VE/1995	AF425638	Venezuela	1995
671.	AF425633/VE/1995	AF425633	Venezuela	1995
672.	AF425635/VE/1995	AF425635	Venezuela	1995
673.	GU056030/VE/1997	GU056030	Venezuela	1997
674.	GU056029/VE/1997	GU056029	Venezuela	1997
675.	AF425634/VE/1997	AF425634	Venezuela	1997
676.	FJ639735/VE/1997	FJ639735	Venezuela	1997
677.	GU056033/VE/1998	GU056033	Venezuela	1998
678.	FJ639741/VE/1998	FJ639741	Venezuela	1998
679.	FJ639740/VE/1998	FJ639740	Venezuela	1998
680.	GU056031/VE/1998	GU056031	Venezuela	1998
681.	GU056032/VE/1998	GU056032	Venezuela	1998
682.	FJ639743/VE/1999	FJ639743	Venezuela	1999
683.	GU131832/VE/2000	GU131832	Venezuela	2000
684.	KF955411/VE/2000	KF955411	Venezuela	2000
685.	GU131833/VE/2000	GU131833	Venezuela	2000
686.	GU131834/VE/2001	GU131834	Venezuela	2001
687.	FJ639802/VE/2004	FJ639802	Venezuela	2004
688.	FJ639794/VE/2004	FJ639794	Venezuela	2004
689.	FJ744701/VE/2004	FJ744701	Venezuela	2004
690.	FJ639797/VE/2004	FJ639797	Venezuela	2004
691.	FJ639796/VE/2004	FJ639796	Venezuela	2004
692.	JN819425/VE/2004	JN819425	Venezuela	2004
693.	GU131835/VE/2004	GU131835	Venezuela	2004
694.	GU131836/VE/2004	GU131836	Venezuela	2004
695.	KF955412/VE/2004	KF955412	Venezuela	2004
696.	GU131837/VE/2005	GU131837	Venezuela	2005
697.	FJ639808/VE/2005	FJ639808	Venezuela	2005
698.	JN819411/VE/2005	JN819411	Venezuela	2005
699.	FJ639813/VE/2005	FJ639813	Venezuela	2005
700.	KF955407/VE/2005	KF955407	Venezuela	2005
701.	JN819412/VE/2005	JN819412	Venezuela	2005
702.	FJ639812/VE/2005	FJ639812	Venezuela	2005
703.	FJ639811/VE/2005	FJ639811	Venezuela	2005
704.	JN819410/VE/2005	JN819410	Venezuela	2005
705.	FJ639814/VE/2005	FJ639814	Venezuela	2005
706.	FJ810415/VE/2005	FJ810415	Venezuela	2005

707.	KF955413/VE/2006	KF955413	Venezuela	2006
708.	HQ332178/VE/2006	HQ332178	Venezuela	2006
709.	HQ332177/VE/2006	HQ332177	Venezuela	2006
710.	JN819413/VE/2006	JN819413	Venezuela	2006
711.	FJ639823/VE/2006	FJ639823	Venezuela	2006
712.	FJ639820/VE/2006	FJ639820	Venezuela	2006
713.	FJ639824/VE/2006	FJ639824	Venezuela	2006
714.	JN819405/VE/2006	JN819405	Venezuela	2006
715.	HQ332180/VE/2006	HQ332180	Venezuela	2006
716.	FJ639818/VE/2006	FJ639818	Venezuela	2006
717.	FJ639821/VE/2006	FJ639821	Venezuela	2006
718.	KF955414/VE/2006	KF955414	Venezuela	2006
719.	FJ639819/VE/2006	FJ639819	Venezuela	2006
720.	FJ639815/VE/2006	FJ639815	Venezuela	2006
721.	GU131838/VE/2006	GU131838	Venezuela	2006
722.	HQ332181/VE/2006	HQ332181	Venezuela	2006
723.	GU131839/VE/2006	GU131839	Venezuela	2006
724.	HQ332182/VE/2006	HQ332182	Venezuela	2006
725.	JN819415/VE/2006	JN819415	Venezuela	2006
726.	FJ639806/VE/2007	FJ639806	Venezuela	2007
727.	JF804026/VE/2007	JF804026	Venezuela	2007
728.	FJ873809/VE/2007	FJ873809	Venezuela	2007
729.	FJ850101/VE/2007	FJ850101	Venezuela	2007
730.	EU482609/VE/2007	EU482609	Venezuela	2007
731.	FJ850099/VE/2007	FJ850099	Venezuela	2007
732.	JN819414/VE/2007	JN819414	Venezuela	2007
733.	HQ332179/VE/2007	HQ332179	Venezuela	2007
734.	FJ882579/VE/2007	FJ882579	Venezuela	2007
735.	GU131842/VE/2007	GU131842	Venezuela	2007
736.	FJ850100/VE/2007	FJ850100	Venezuela	2007
737.	EU482610/VE/2007	EU482610	Venezuela	2007
738.	GU131840/VE/2007	GU131840	Venezuela	2007
739.	FJ850102/VE/2007	FJ850102	Venezuela	2007
740.	GQ199877/VE/2007	GQ199877	Venezuela	2007
741.	EU482611/VE/2007	EU482611	Venezuela	2007
742.	KF955441/VE/2007	KF955441	Venezuela	2007
743.	GU131841/VE/2007	GU131841	Venezuela	2007
744.	FJ873810/VE/2007	FJ873810	Venezuela	2007
745.	HQ332183/VE/2007	HQ332183	Venezuela	2007
746.	FJ850104/VE/2008	FJ850104	Venezuela	2008
747.	FJ850103/VE/2008	FJ850103	Venezuela	2008

Table S2. Complete dataset, subsets Clade I and Clade II of Genotype V (clade cosmopolitan) DENV-1.

Location	Country / Sampling dates	Complete dataset Genotype V (clade cosmopolitan)	Subset Clade I	Subset Sub-Clade Ia	Subset Sub-Clade Ib	Subset Clade II
**AR**	Argentina / 2000- 2016	115	65	62	2	46
**BR**	Brazil / 1982 - 2016	171	48	28	20	67
BRCO	*Brazil: Central-Western*	*27*	*4*	*4*	-	*17*
BRSE	*Brazil: Southeastern*	*62*	*18*	*18*	-	*21*
BRS	*Brazil: South*	*1*	-	-	-	-
BRNE	*Brazil: Northeastern*	*38*	*7*	*6*	*1*	*10*
BRN	*Brazil: Northern*	*42*	*19*	-	*19*	*19*
	No information	*1*	-	-	-	-
**AW**	Aruba / 1985-2004	2	-	-	-	-
**BB**	Barbados / 1995-2003	5	-	-	-	-
**BS**	Bahamas / 1977	1	-	-	-	-
**BZ**	Belize / 2005	1	-	-	-	-
**CN**	China / 2016	1	1	1	-	-
**CO**	Colombia / 1985-2008	20	18	-	-	-
**CR**	Costa Rica / 1993-2005	4	-	-	1	-
**DO**	Dominican Republic / 2007	1	1	-	-	-
**GD**	Granada / 1977-1981	9	-	-	-	-
**GF**	French Guiana / 1989	1	-	-	-	-
**GY**	Guyana / 2008	1	1	-	1	-
**HN**	Honduras / 2008	1	-	-	-	-
**HT**	Haiti / 2010	5	5	-	-	-
**IN**	India / 1962-2007	13	-	-	-	-
**JM**	Jamaica / 1977	1	-	-	-	-
**MQ**	Martinique / 1989-2008	4	3	-	-	-
**MX**	Mexico / 1980 - 2012	149	-	-	-	-
**NI**	Nicaragua / 1996 - 2009	51	-	-	-	-
**PE**	Peru / 1991	1	-	-	-	-
**PR**	Puerto Rico / 1986-2012	64	27	-	-	1
**PY**	Paraguay / 1999-2000	5	-	-	-	-
**RE**	Reunion Island / 2004	2	-	-	-	-
**SB**	Saint Barthelemy /2016	1	-	-	-	-
**SG**	Singapore / 1990 - 2005	2	-	-	-	-
**SR**	Suriname / 1981	1	-	-	-	-
**SV**	El Salvador / 1993 - 2012	5	-	-	-	-
**TT**	Trinidad and Tobago / 1978-1986	3	-	-	-	-
**US**	United States / 2009 - 2014	24	5	-	-	-
**VE**	Venezuela / 1994-2008	81	66	15	32	-
**VG**	British Virgin Islands / 1985-1987	2	-	-	-	2
**Total**		**747**	**240**	**106**	**56**	**116**
